# Global Study of Plant‐Herbivore Interactions Reveals Similar Patterns of Herbivory Across Native and Non‐Native Plants

**DOI:** 10.1111/ele.70196

**Published:** 2025-08-14

**Authors:** Andrea Galmán, Philip G. Hahn, Brian D. Inouye, Nora Underwood, Yanjie Liu, Susan R. Whitehead, William C. Wetzel

**Affiliations:** ^1^ Department of Land Resources & Environmental Sciences Montana State University Bozeman Montana USA; ^2^ Entomology and Nematology Department University of Florida Gainesville Florida USA; ^3^ Department of Biological Science Florida State University Tallahassee Florida USA; ^4^ Northeast Institute of Geography and Agroecology, Chinese Academy of Sciences Changchun China; ^5^ Department of Biological Sciences Virginia Polytechnic Institute and State University Blacksburg Virginia USA

**Keywords:** ecological factors, ecological novelty, intra‐population variability, non‐native plants, plant‐herbivore interactions

## Abstract

A core hypothesis in invasion and community ecology is that species interaction patterns should differ between native and non‐native species due to non‐native species lacking a long evolutionary history in their resident communities. Numerous studies testing this hypothesis yield conflicting results, often focusing on mean interaction rates and overlooking the substantial within‐population variability in species interactions. We explored plant‐herbivore interactions in populations of native and established non‐native plant species by quantifying differences in mean herbivory and added a novel approach by comparing within‐population variability in herbivory. We include as covariates latitude, plant richness, plant growth form and cover. Using leaf herbivory data from the Herbivory Variability Network for 788 plant populations spanning 504 species globally distributed, we found no overall differences in mean herbivory or variability between native and non‐native plants. These results suggest native and established non‐native plants interact similarly with herbivores, indicating non‐native status is not a strong predictor of ecological roles.

## Graphical abstract

We conducted a global comparison of herbivory between native and non‐native species, using data from 788 populations of 504 plant species. After accounting for differences in introduction status, time since introduction, and several ecological factors, we found no differences in mean herbivory or variability in herbivory between native and non‐native plants.

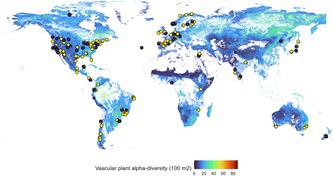

## 1 Introduction

The establishment of non‐native species in novel environments contributes to global change by altering biotic interactions and ecosystem functioning (Bellard et al. 2022; Vilà et al. 2011). Unlike natives, non‐natives lack a long coevolutionary history with the organisms in their introduced range. The idea that a lack of evolutionary history influences interactions with other organisms has a long history in ecology (Darwin 1859). For example, the Enemy Release Hypothesis predicts less herbivore damage on recently introduced plants because herbivores have not evolved to recognise them as hosts (Keane and Crawley 2002; Williamson 1996). Indeed, reduced herbivory is a clear driver of success in multiple invasions (Liu and Stiling 2006; Meijer et al. 2016), and herbivores have been used as successful biocontrol agents in important systems (Malecki et al. 1993; Hultine et al. 2010). In contrast, studies of specific clades or habitats have found non‐natives with similar or elevated mean levels of herbivory (Agrawal and Kotanen 2003; Agrawal et al. 2005; Colautti et al. 2004; Pearse and Hipp 2009). These findings have led to debate about the importance of non‐native status as a general predictor of ecological interaction patterns (Carrillo‐Gavilán et al. 2012; Ivison et al. 2023; Lundgren et al. 2024), whereas others have advocated for studies to examine covariates that could mask differences between natives and non‐natives (Brian and Catford 2023; Catford et al. 2022; Chiuffo et al. 2022). Here, we use a standardised global survey to test the novel hypothesis that a key component of differences in plant‐herbivore interactions between native and established non‐native plants could be differences in the variability of herbivory among plant individuals within populations. Whereas past studies have focused on mean herbivory, an explicit examination of interaction variability could help reveal whether non‐native plants interact with herbivores in ecologically different ways than native plants.

Variability is a critical feature of biological systems, and ecologists are increasingly recognising the important role it plays in shaping the outcome of competition, consumer‐resource interactions and population dynamics (Benedetti‐Cecchi 2003; Bolnick et al. 2011; Holyoak and Wetzel 2020; Inouye 2005; Shoemaker et al. 2020; Violle et al. 2012; Wetzel et al. 2023). For example, theory indicates that factors that increase the variability of herbivore attack among individuals within plant populations should stabilise plant‐herbivore interactions and reduce the probability of plant extirpation (Anderson and May 1978; Bjørnstad and Hansen 1994; Crawley 1983). Indeed, when herbivory is aggregated only on a few plant individuals within a population, many plants can escape top‐down pressure from herbivores; thus, a higher equilibrium population size can be achieved (Anderson and May 1978; Crawley 1983). Aggregation also means that herbivores may experience greater negative density‐dependent feedback, which can stabilise host‐herbivore population dynamics (Mutz and Inouye 2023). Thus, for similar levels of mean herbivory, the impact of herbivores might be greater in a population where all plant individuals are attacked to a similar extent than in a population that exhibits high variability in herbivory attack. This suggests that different herbivore impacts could occur if herbivore damage were more variable in populations of non‐natives than natives, even if the mean amounts of herbivore damage across populations were similar. Regardless of their potential consequences, differences in intrapopulation variability in herbivory between native and non‐native species would indicate differences in interactions with herbivores.

A key factor likely influencing intrapopulation variability in herbivory is how plants are recognised by their potential enemies (Wetzel et al. 2023). Non‐native and native species could therefore differ in their levels of intrapopulation variation in herbivory if their main herbivores differed in host‐plant recognition or acceptance behaviours. Specialist and generalist herbivores may differ in the cues they use for host‐plant recognition, and herbivores belonging to different taxonomic groups may differ in host recognition and inter‐plant movement behaviours. For example, non‐natives would exhibit higher variability in herbivory if most non‐native individuals are not recognised by herbivores owing to their novelty, but those few individuals that are recognised (or sampled) by herbivores suffer high damage. High damage on the few unlucky individuals that are used as hosts might be expected because many non‐native species prioritise growth over defence (Fahey et al. 2022; Huang et al. 2020; van Kleunen et al. 2010). Alternatively, non‐natives could exhibit lower variability in herbivory than natives because they are less likely to be recognised as hosts by specialists than by generalist herbivores (Goßner et al. 2009; Parker and Hay 2005; Parker et al. 2006). Specialists often have patchy distributions, potentially leading to more variable damage (Price 2003) on natives, whereas the generalist‐dominated herbivore community on non‐natives may leave more homogeneous damage (Joy Massad et al. 2024). Finally, non‐native status might be a poor predictor of host recognition by herbivores, leading to no overall differences in variability in herbivory between natives and non‐natives. This result would support the perspective that non‐native status is a poor predictor of ecological roles relative to a species' traits (Agrawal and Kotanen 2003; Lundgren et al. 2024).

A key recognition from the literature testing differences in herbivory between native and non‐native plants over the last decade is that herbivore recognition of non‐native plants can vary with geographic and ecological context, both of which can greatly influence on species interactions (Brian and Catford 2023; Catford et al. 2022; Chiuffo et al. 2022; Gioria et al. 2023; Xu et al. 2021). For example, recent studies testing the hypothesis that plant‐herbivore interactions vary in a different way with latitude for native and non‐native plants (i.e., non‐parallel latitudinal gradients in herbivory for natives and non‐natives) have yielded contrasting results (Allen et al. 2017; Bezemer et al. 2014; Cronin et al. 2015). Some studies focusing on model species found that differences in mean herbivory between natives and non‐natives are more likely to be found in lower latitudes (where there is higher richness of specialised herbivores that attack mostly non‐native species—Dyer et al. 2007; Mittelbach et al. 2007) and the difference between both groups gets weaker with increasing latitude (as the abundance of generalist herbivores that attack natives and non‐natives increases; Bezemer et al. 2014; Guo et al. 2024). However, a recent global study reported no differences in latitudinal patterns of herbivory for both groups of plants (Xu et al. 2021). We argue that there are two important gaps in this literature. First, these studies only examined latitude and not other important factors that influence plant‐herbivore interactions, such as plant diversity, growth form or plant cover. Second, ecological context should influence how host recognition of non‐native species affects variability in herbivory as well as mean levels of herbivory. Past studies of geographical and ecological variation have only considered mean levels of herbivory; however, the Herbivory Variability Network (2023) found that variability in herbivory showed an even greater increase with latitude than did mean levels of herbivory.

The amount and variability of herbivory on plants are influenced by herbivore recognition, which is shaped by ecological factors. We identify two mechanisms by which ecological factors might influence herbivore recognition of non‐native species. First, herbivore recognition might depend on factors such as latitude and regional plant diversity, which are predictors of herbivore abundance and richness (Schemske et al. 2009; Zhang et al. 2016). In environments with high herbivore abundance and richness (such as high plant diversity environments or tropical regions), non‐native plants might have a higher risk of being detected and attacked by one or more herbivore species, leading to high mean herbivory for both natives and non‐natives (i.e., an increase in species diversity may increase attack risk; Schuldt et al. 2015). However, non‐native plants may have lower mean herbivore attack rates than natives in environments with low herbivore richness (such as low plant diversity environments or temperate regions). We predict that non‐natives will exhibit higher variability in herbivory than natives in environments with high herbivore abundance and richness, because natives should be consistently attacked by their generalist and specialist herbivores while non‐natives will either be overlooked or heavily attacked when found (mostly by generalist herbivores, but potentially by some specialist species). Second, differences in plant‐herbivore interactions between native and non‐native plants might depend on plant characteristics that influence how easily a plant is detected by herbivores, such as growth form or percent cover (Plant Apparency; Feeny 1976; Galmán et al. 2018; Strauss et al. 2015). Plants with characteristics that make them more obvious hosts for herbivores (woody species or high‐cover plants) should have higher levels of attack regardless of plant status, but within a population, non‐native plants should have higher variability in herbivory than natives because natives should be attacked more consistently by a greater number of herbivore species.

Understanding how non‐native plants interact with the herbivores in their introduced range requires work that examines how factors such as latitude, plant diversity, plant growth form, or plant cover influence both the mean and variability of herbivory in native and non‐native populations. Previous large‐scale studies only investigated a limited number of plant species (but see Xu et al. 2021) and data for these studies were obtained using different methods, making comparisons across systems difficult (Meijer et al. 2016). Here, we perform a broad global comparison of plant‐herbivore interaction patterns between native and non‐native species using the largest dataset thus far to address this question and using a common protocol across all species. We use data from surveys conducted by the Herbivory Variability Network (herbvar.org). Our study is the first to compare mean and variability in herbivory between native and non‐native plants. We conduct (i) a large biogeographical analysis across 788 populations (616 native and 172 introduced) of 504 plant species; and (ii) an analysis comparing native and introduced ranges in a subset of ten species for which we collected survey data from both parts of their range. For each objective, we ask (1) if there are broad differences in mean herbivory and variability in herbivory between native and non‐native populations of many species; and (2) if those differences are modulated by ecological context, such as herbivore abundance and richness (using the proxies latitude and plant richness) and factors modulating the interaction between plants and herbivores (plant growth form and focal plant cover).

## 2 Methods

### 2.1 Field Surveys

**FIGURE 1:**
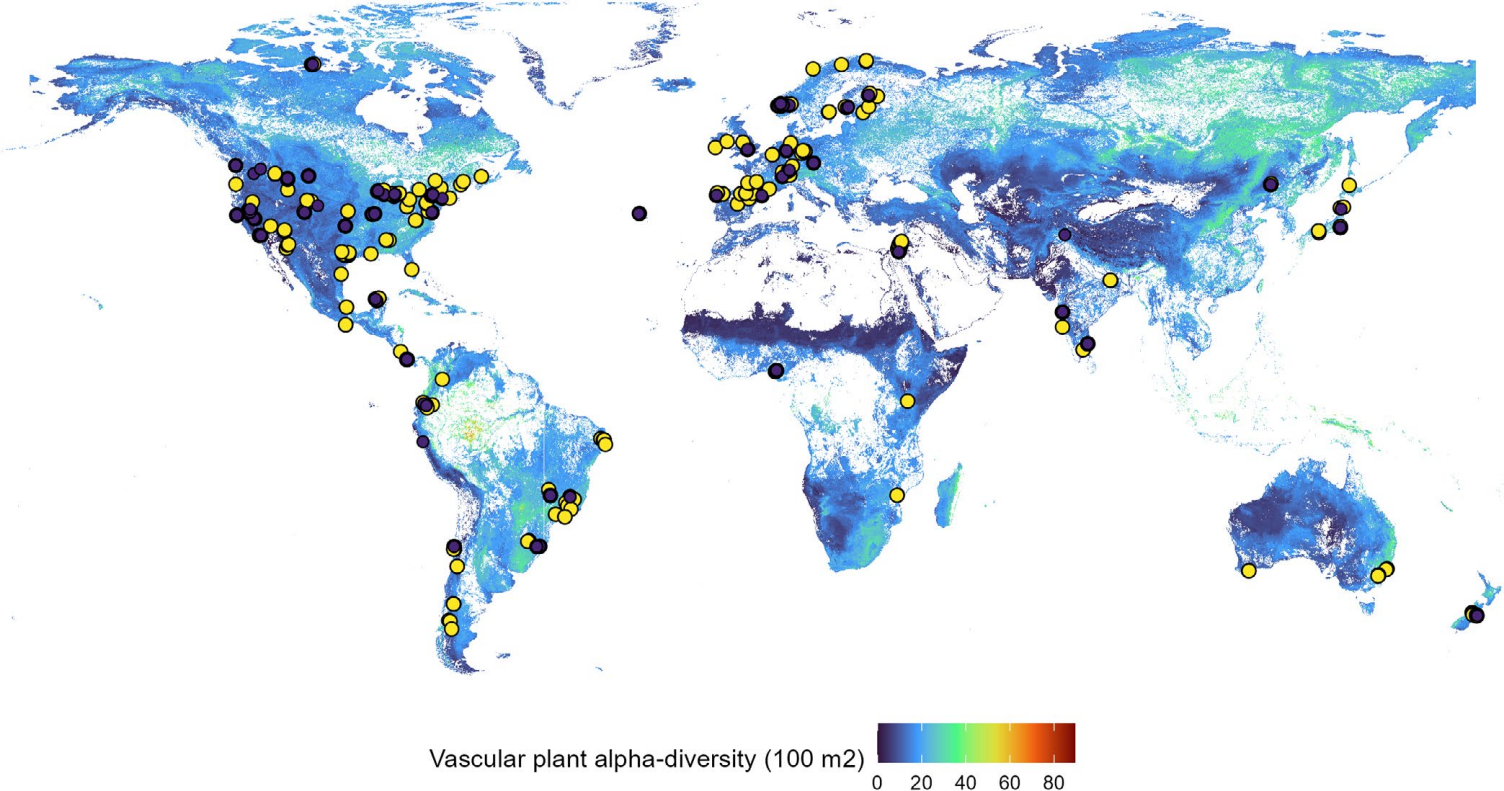
Geographic and environmental locations for the native (yellow dots, *n* = 616) and non‐native (purple dots, *n* = 172) populations of the studied species. The map shows the global distribution of estimated vascular plant alpha diversity (spatial grain 100 m^2^).

We used data from the Herbivory Variability Network (herbvar.org), a team of researchers from 34 countries focused on the role of variability in plant‐herbivore interactions. The dataset includes surveys of aboveground herbivory in 788 plant populations (616 native, 172 non‐native) encompassing 504 species from 135 families across six continents (Figure 1). Collaborators selected sampling dates at biologically important times of the growing season, based on species and habitat.

Collaborators followed a standardised protocol (Data S1). For each survey, we randomly selected 30 individuals and their nearest conspecific neighbours, resulting in a total of 60 plants. Our large sample size allows for a robust estimation of within‐population variability, as well as mean herbivory. For each plant, we visually estimated the aboveground proportion of herbivore damage, following a detailed guide. We included all visible damage from invertebrates and vertebrates. For plants under 2 m tall, we examined all aboveground tissue; for larger plants, we subsampled 30 leaves. We also recorded the percent cover of the focal plant species in circular quadrats around each individual (with radius ranging from 0.4–3.6 m, depending on the size of the plant, Data S1). Alternative protocols cover species with specific characteristics, such as clonal plants, low‐abundance plants, trees, and succulents or cacti.

### 2.2 Data Acquisition

We classified populations as native or non‐native based on information provided by the local scientific collaborator and corroborated when needed with the Plants of the World Online database (POWO 2024). We checked the naturalisation status of non‐native species in Global Naturalised Alien Flora (GloNAF; van Kleunen et al. 2019). Most non‐native species in our study are naturalised and widespread in their introduced ranges; only seven populations (of six non‐native plant species) are not naturalised (Table S1 in Supporting Information). We also extracted information on invasiveness from the CABI Digital Library (cabidigitallibrary.org) where species were surveyed. When invasiveness was unavailable in CABI, we consulted regional repositories. Of 172 non‐native populations, 112 were locally invasive while 37 were not (Table S1). We collected non‐native introduction dates by using the earlier of the first regional observation in Global Biodiversity Information Facility (GBIF; gbif.org) and the first mention of the species in the region in the primary literature (Table S1).

We extracted plant species richness (estimated number of plant species per 100 m^2^) for our study sites from sPlot, which predicts plant diversity combining global vegetation surveys and mathematical models (Bruelheide et al. 2019; Sabatini et al. 2022). Since sPlot does not provide uncertainty measures for the estimates of plant diversity, we do not include those in our analyses; thus all results for plant diversity should be interpreted cautiously.

### 2.3 Statistical Analyses

For our analyses, we used mean herbivory within a population and variability in herbivory among individuals within a population as separate response variables. We calculated mean herbivory as the average proportion of damage across individuals in a population. For each population, we summarised the variability in herbivory across individuals by calculating the Gini coefficient, using the R package DescTools (Signorell et al. 2019). The Gini coefficient of variability (range 0–1) describes the level of unevenness or inequality among units. The Gini coefficient is calculated with L‐moments instead of conventional moments, making it less sensitive to outliers and more reliable at small sample sizes (Valbuena et al. 2017).

We fit Bayesian phylogenetic generalised linear mixed models (GLMM) in the brms package (Bürkner 2021) in R version 4.3.0 (R Core Team 2023), with beta response distribution (well suited to represent variables on the 0–1 interval; Douma and Weedon 2019). Since the beta distribution is undefined for 0 and 1, we truncated three values in our data to 0.99; no populations had a Gini coefficient of zero. Models ran across seven MCMC chains for at least 5 000 iterations. Here, and for the GLMMs for other questions, we assessed runs by ensuring all R‐hat values were < 1.03, and visually checked fits via posterior predictive checks. For prior distributions we used Normal (0, 2) for slopes, Normal (0, 2) for intercepts, gamma (1, 0.05) for phi [the beta distribution dispersion parameter] and Cauchy (0, 1) for the standard deviation of random effects. We account for phylogenetic correlations using a phylogenetic tree for the species in our study with the phylo.maker function in ‘V.PhyloMaker’ (Jin and Qian 2019) and ‘ape’ (Paradis and Schliep 2019) R‐packages by matching the family, genus, and species epithet from our survey with those in the backbone using the GBOTB.extended phylogeny (i.e., the mega‐tree implemented in the ‘V.PhyloMaker’ R package). For each model, we report effect sizes, 95% credible intervals (CIs), Bayes Factors (BF) and marginal Bayesian *R* ^2^ values.

#### 2.3.1 Global Differences in Mean Herbivory and Variability in Herbivory Between Native and Non‐Native Populations

We compare herbivory between native and non‐native plant populations of different species globally distributed and between populations of the same species in their native and non‐native ranges. We ran Bayesian phylogenetic GLMMs using mean herbivory or the Gini coefficient of herbivory as response variables and plant invasive status (i.e., native, invasive or not invasive non‐native) as a fixed effect. For the global dataset (788 populations, 504 species), we include plant species and phylogeny as random effects. We ran the same models (without the plant phylogeny) for the biogeographical subset of ten species (Table S2 in Supporting Information) occurring in native and introduced ranges, using plant status (native or non‐native) as a fixed effect and included the interaction between range and species.

For the global dataset, we ran two additional models using plant naturalisation status (i.e., native, naturalised or not naturalised non‐native) or plant status (i.e., native, non‐native) as fixed effects. Due to the small sample size for non‐naturalised populations and the lack of significant differences between natives or naturalised non‐natives, we show the results for the analyses with naturalised status only in the Supporting Information material (Figure S1).

#### 2.3.2 Effects of Ecological Context

To test whether differences in herbivory were contingent on ecological factors, we again ran Bayesian phylogenetic GLMMs using the Gini coefficient or mean herbivory as response variables. For the global dataset (788 populations, 504 species), we included plant invasive status (i.e., native, invasive or not invasive non‐native) as a fixed factor and its interactions with (i) plant diversity, (ii) latitude (absolute values), (iii) focal plant cover and (iv) growth form (woody vs. non‐woody species). We included plant species and plant phylogeny as random effects. We also ran models with naturalisation status (i.e., native, naturalised or not naturalised non‐native) and plant status (i.e., native or non‐native) as fixed effects (Figure S2). For the interactions with plant diversity and plant cover, we ran the same models for the biogeographical subset of ten populations occurring in native and introduced ranges, including the interaction with species as a fixed effect.

#### 2.3.3 Effect of the Time Since Introduction for Non‐Native Species

To test whether mean herbivory and variability in herbivory for non‐native populations depend on their introduction time, we ran a Bayesian phylogenetic GLMM using the Gini coefficient or mean herbivory as response variables. We used the year of introduction (earliest year the species was reported in the sampled region) as a fixed factor, and we included plant species and plant phylogeny as random effects.

## 3 Results

### 3.1 Global Differences in Mean Herbivory and Variability in Herbivory Between Native and Non‐Native Populations

**FIGURE 2:**
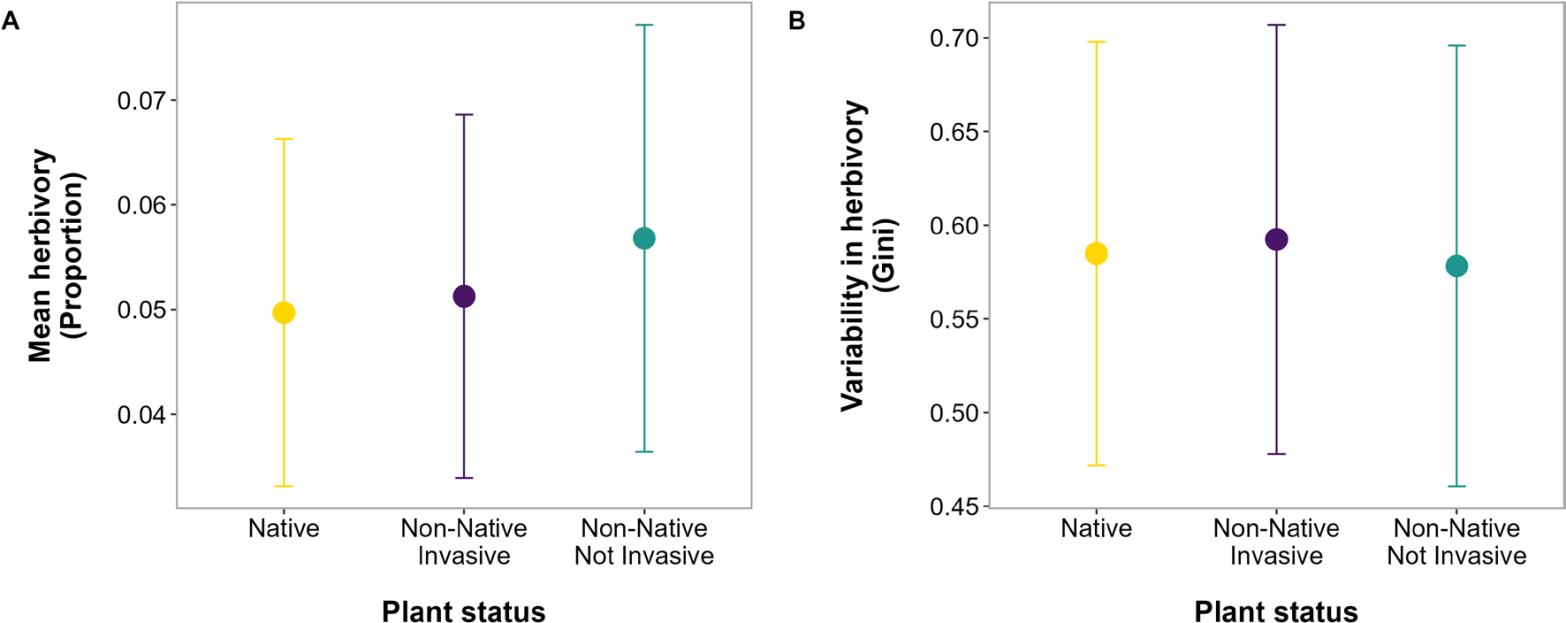
Results from the global analysis of enemy release showing no differences in (A) mean herbivory and (B) variability in herbivory (Gini coefficient) between native (yellow), invasive (purple) and not invasive (green) non‐native species. Dots show predicted means and lines 95% credible intervals from Bayesian phylogenetic beta regressions.

Mean herbivory and variability in herbivory were similar between native and non‐native species across the globe, independently of the invasive status of non‐natives. Mean herbivory averaged 5% for both native (95% CI = 1.9%–12%) and invasive non‐native species (95% CI = 2.4%–11%) and 6% for non‐invasive non‐native species (95% CI = 1.9%–15%, *R* ^2^ = 6%, BF = 0.1, Figure 2a). Gini coefficients were 0.58 (0.31–0.8) for native, 0.59 (0.28–0.82) for non‐invasive, and 0.60 (0.36–0.79) for invasive non‐native species (*R* ^2^ = 5%, BF = 0.09, Figure 2b).

Results were similar when comparing species' native and non‐native status without considering their invasive status (Figure S3). Non‐native status also had no effect on the mean or variability of herbivory when we restricted our dataset to the ten species with surveys in native and introduced ranges (Figures S4 and S5).

### 3.2 Effect of Ecological Context: Latitude

**FIGURE 3:**
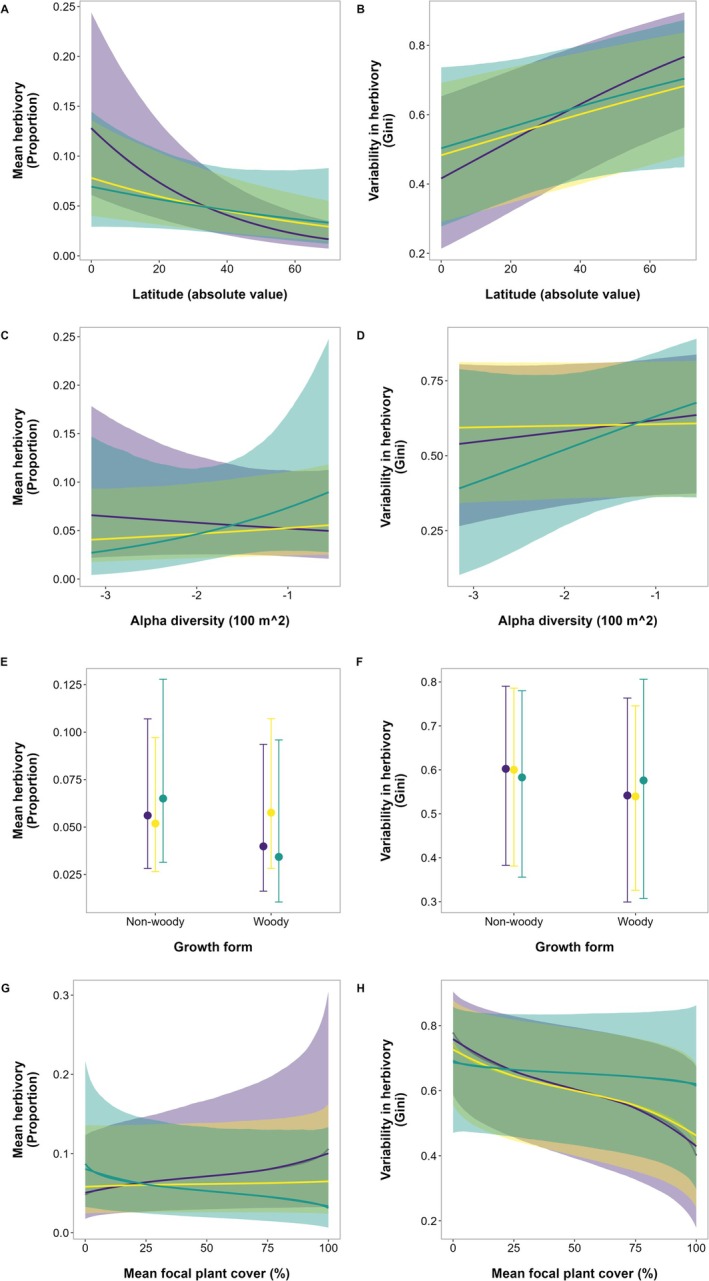
Results from the analyses of ecological factors potentially driving differences in herbivory for native (yellow), invasive (purple) and not invasive (green) non‐native species populations. There were no differences in mean herbivory or variability in herbivory between native and non‐native populations at different latitudes (A, B), different levels of plant alpha diversity (C, D), nor considering different growth forms (E, F) or with different focal plant cover (G, H). Graphs show predicted means and 95% credible intervals from Bayesian phylogenetic beta regressions.

Mean herbivory decreased with increasing latitude from 13% (95% CI: 6%–24%) at the equator to 2% (95% CI: 1%–4%) at 70° N/S (*R* ^2^ = 6%, BF = 17.9, Figure 3a). In contrast, variability in herbivory increased with increasing latitude from Gini = 0.40 (95% CI: 0.2–0.7) at the equator to Gini = 0.80 (95% CI: 0.6–0.9) at 70° N/S (*R* ^2^ = 5%, BF = 1.2, Figure 3b). The data did not support an interaction between invasive status (i.e., native, invasive and not invasive non‐natives) and latitude for mean damage (Estimate = 0.02, 95% CI = 0–0.04, *R* ^2^ = 6%, BF = 0.03, Figure 3a) or for variability in herbivory (Estimate = −0.01, 95% CI = −0.03 to 0.01, *R* ^2^ = 5%, BF = 0.01, Figure 3b).

Results were similar when comparing native and non‐native status, without considering the invasive status of non‐natives (Figure S6).

### 3.3 Effect of Ecological Context: Plant Diversity

Neither mean herbivory (Estimate = −0.12, 95% CI = −0.56 to 0.34, *R* ^2^ = 6%, BF = 0.13, Figure 3c) nor variability in herbivory (Gini = 0.15, 95% CI = −0.23 to 0.52, *R* ^2^ = 5%, BF = 0.13, Figure 3d) were related to plant diversity; herbivory was similar for regions with low diversity (4 plant species per 100 m^2^) and the highest diversity (37 plant species per 100 m^2^). The data did not support an interaction between invasive status (i.e., native, invasive and not invasive non‐natives) and diversity for mean damage (Estimate = 0.60, 95% CI = −0.40 to 1.61, *R* ^2^ = 6%, BF = 0.51, Figure 3c) or for the Gini coefficient (Estimate = 0.30, 95% CI = −0.59 to 1.19, *R* ^2^ = 5%, BF = 0.30, Figure 3d), suggesting there is no effect of local plant diversity on differences in herbivory between native and non‐native plants.

Results were similar when comparing native and non‐native status, ignoring the invasive status of non‐natives (Figure S6). When comparing native and introduced ranges for the subset of ten species, we again found no relationship between plant diversity and patterns of mean or variability of herbivory (Figures S7 and S8).

### 3.4 Effect of Ecological Context: Plant Growth Form

There was no effect of growth form on mean herbivory (Estimate = −0.40, 95% CI = −1.05 to 0.34, *R* ^2^ = 6%, BF = 0.29, Figure 3e) or variability in herbivory (Gini = −0.25, 95% CI = −0.81 to 0.30, *R* ^2^ = 5%, BF = 0.21, Figure 3f), or interactions with growth form (Mean = −0.33, 95% CI = −1.57 to 0.87, *R* ^2^ = 6%, BF = 0.34, Figure 3e; Gini = 0.22, 95% CI = −0.75 to 1.17, *R* ^2^ = 5%, BF = 0.27, Figure 3f) for native, invasive, and not invasive non‐native species. Results were similar when analysing the data comparing only native or non‐native status (Figure S6).

### 3.5 Effect of Ecological Context: Focal Plant Cover

There was no effect of focal plant cover on mean herbivory (Estimate = 0.12, 95% CI = −0.07 to 0.30, *R* ^2^ = 5%, BF = 0.11, Figure 3g) but we found that variability was lower, indicating a more even distribution of herbivory, when focal plant cover was higher (Gini = −0.23, 95% CI = −0.38 to −0.08, *R* ^2^ = 4%, BF = 3.72, Figure 3h). There were no interactions between focal plant cover and invasive status (i.e., native, invasive and not invasive non‐natives) for either response variable (Mean = −0.27, 95% CI = −0.56 to 0.03, *R* ^2^ = 5%, pp. = 1, BF = 0.36, Figure 3g; Gini = 0.18, 95% CI = −0.06 to 0.42, *R* ^2^ = 4%, pp. =1, BF = 0.18, Figure 3h). These results, plus the fact that non‐native cover was only 7% greater than native cover on average, suggest that plant cover influences herbivory in similar ways for native and non‐native plants.

Results were similar when analysing the data comparing native and non‐native status (Figure S6). When comparing native and introduced ranges for the subset of ten species, we did not find any effect of cover on either response variable (Figures S9 and S10).

### 3.6 Effect of the Time Since Introduction for Non‐Native Species

We found no differences in mean herbivory (Estimate = −3.15; 95% CI = −5.95 to −0.60; R^2^ = 5%, pp. = 1, BF = 0.01, Figure S11) or variability in herbivory (Gini = 1.76; 95% CI = −0.01 to 0; *R* ^2^ = 2%, pp. = 1, BF = 0.01, Figure S11) based on the year of introduction of the non‐native species. The earliest year of introduction reported for our species was 1500 and the latest was 2000. Most non‐native species in our dataset were introduced in the 19th and 20th centuries.

## 4 Discussion

Using a global standardised survey of herbivore damage on 788 populations of 504 plant species, we evaluated whether mean herbivory differs between native and non‐native plants. We proposed a novel approach to test differences in plant‐herbivore interactions between native and non‐native plants by exploring potential differences in the variability of herbivory and by including the effects of several biotic and abiotic factors potentially masking differences in herbivory. Despite this expanded perspective and the breadth and intensity of our sampling, we found no differences in mean damage or variability in herbivory between natives and non‐natives, even when including the invasive status or time of introduction of the non‐native populations, suggesting that there are no overall differences in plant‐herbivore interactions based on plant status. Below we discuss potential explanations for this lack of differences in herbivory patterns and the implications of this finding for invasion ecology.

Previous studies suggested that differences in herbivore recognition between natives and non‐natives may emerge only after accounting for key ecological covariates that influence herbivory (Brian and Catford 2023; Catford et al. 2022; Chiuffo et al. 2022; Gioria et al. 2023). However, after we accounted for variation in herbivory with latitude, local plant species richness, growth form, and focal species percent cover—all factors that can be key determinants of herbivory—we still found no differences in herbivore damage between native and non‐native populations. While latitude and focal species cover had strong relationships with the mean and variability of herbivory on non‐native populations, these relationships were strikingly similar to those for native plants. This result suggests that, on average, established non‐native species might be more ecologically similar to natives in plant‐herbivore interactions than previously thought. The similarity in the relationship between local abundance (percent cover) and variability in herbivory for both groups suggests that ecological factors like abundance influence herbivore host recognition and use more than plant status. Low‐abundance, locally rare populations may experience high variability in damage—rare individuals either escape notice or are found and highly consumed—regardless of their plant status. Overall, the broad ecological factors we examined appear to be stronger predictors of herbivory patterns than non‐native status, aligning with recent studies showing that non‐native status is a poor predictor of ecological roles (e.g., Lundgren et al. 2024).

One explanation for the lack of differences in herbivory between native and non‐native plants is that the interaction between non‐native plants and native herbivores is determined by the specific plant traits and their functional similarity to the native community, rather than by their non‐native status. Indeed, previous studies have shown that phylogenetic relatedness and trait similarity to native species predict herbivory in non‐native species (Pearse and Hipp 2009; Pearse and Rosenheim 2020). Studies on invasion ecology have also highlighted the predominant role of functional traits for the success of non‐native species (El‐Barougy et al. 2020; Qian and Sandel 2022), indicating that non‐native species that are functionally similar to natives benefit from preadaptation to the novel environment and are more likely to naturalise. One implication of the conclusion that interactions with native herbivores depend on the non‐native traits is that the lack of herbivore recognition for non‐natives is not widespread. More likely, only a fraction of non‐native species benefit from a reduced attack by herbivores when introduced into a novel environment due to differences in host detection, depending on the environment and the specific traits of the species. Indeed, reduced herbivore attack may be important only for the establishment of those rare non‐native species that differ significantly in traits from their native competitors. Alternatively, combined with work reporting lower insect diversity feeding on non‐native plants (Burghardt et al. 2009), our results suggest that the native herbivores that recognise and feed upon non‐native plants may feed heavily when switching hosts. Consequently, non‐native plants may be consumed as much as natives but by fewer arthropod species.

A key implication of our finding that herbivory patterns are similar between natives and established non‐natives is that altered herbivore attack via enemy release—though potentially important in early invasion stages—loses relevance once non‐native populations are established, as is the case for the non‐native species in our study. A waning of reduced herbivore recognition is perhaps not surprising as native herbivores have been found to adapt rapidly to use non‐native hosts as a resource (Ivison et al. 2023; Parker and Gilbert 2004; Mitchell et al. 2010). Indeed, we found no differences in herbivory between non‐native species based on their introduction time. Similarly, Xirocostas et al. (2023) reported that herbivory was independent of time since introduction, though they reported a trend towards lower herbivory on non‐native species. In addition, some non‐native plants are introduced together with non‐native herbivores that also naturalise in the introduced range (Brockerhoff and Liebhold 2017; Johnson et al. 2019); it is important to disentangle the respective roles of native and non‐native herbivores to fully understand patterns of herbivory on non‐native plants. Future studies could dive further into this hypothesis by examining herbivory patterns or experimentally excluding or adding herbivores in populations in the initial stages of invasions. It is important to note that our study focuses on single‐time‐period snapshots; factors like timing of damage and leaf ontogeny significantly influence the impact of herbivory on plant fitness (Marquis 1992), and future studies might help understand the impacts of herbivory by increasing the sampling breadth. Regardless, our data provide a robust view of herbivory on established non‐natives across a broad sampling of geography, growth form, and taxonomy.

Overall, our results indicate that established non‐natives integrate into plant‐herbivore interactions in their exotic ranges, indicating that established non‐native species do not escape herbivore recognition in the novel environment based on a lack of a long evolutionary history. Other mechanisms, other than higher resistance to herbivory, may confer non‐native species with competitive advantages over natives. For instance, non‐native species may exhibit greater tolerance to herbivore attack. In this regard, invasive plants have been found to perform better than natives under similar damage levels (Ashton and Lerdau 2008), suggesting that even when non‐native species do not benefit from reduced attack, they might have different mechanisms than natives to tolerate herbivory (Liao et al. 2014), which could help them outcompete native plants. Alternatively, other studies have suggested that arbuscular mycorrhizal fungi and soil nitrogen levels may be critical in mediating the success of introduced plants (Zhang et al. 2023).

Finally, the fact that herbivore damage is not generally lower for non‐natives than natives suggests that introducing herbivores for biocontrol may not effectively manage established non‐native species, in line with the observation that controlling non‐native plants via classical biocontrol is challenging (Shen et al. 2023). Management strategies must be specific to the herbivore community and the traits of the non‐native species. Managers should not rely only on theory to guide their decisions, but will also need to use experimental ecology to elucidate the factors that determine the success of particular non‐native species. In cases where herbivores are used as management tools, our results suggest that successful non‐native management via herbivores could be thought of as increasing herbivory on non‐natives above what is natural, rather than restoring enemy pressure that may not have been escaped in the first place.

## Author Contributions

A.G. and W.C.W. conceptualised the study. All the authors performed the research and collected the data. A.G. analysed the data and wrote the original draft. All the authors contributed substantially to revisions and edited the manuscript. W.C.W. curated the data. The funding acquisition was led by W.C.W. in collaboration with P.G.H., B.D.I., N.U. and S.R.W.

## Peer Review

The peer review history for this article is available at https://www.webofscience.com/api/gateway/wos/peer‐review/10.1111/ele.70196.

## Supporting information


**Data S1:** ele70196‐sup‐0001‐DataS1.pdf.

## Data Availability

Data and Code are available in Dryad at https://doi.org/10.5061/dryad.2jm63xt1w.
