# Supplementary material for: Global Study of Plant‐Herbivore Interactions Reveals Similar Patterns of Herbivory Across Native and Non‐Native Plants
Source: Ecol Lett. 2025 Aug 14;28(8):e70196. doi: 10.1111/ele.70196 (PMC12352374; doi:10.1111/ele.70196)
Supplement: Supplementary file 1 — Data S1: ele70196‐sup‐0001‐DataS1.pdf. [file ELE-28-0-s001.pdf]

SUPPORTING INFORMATION

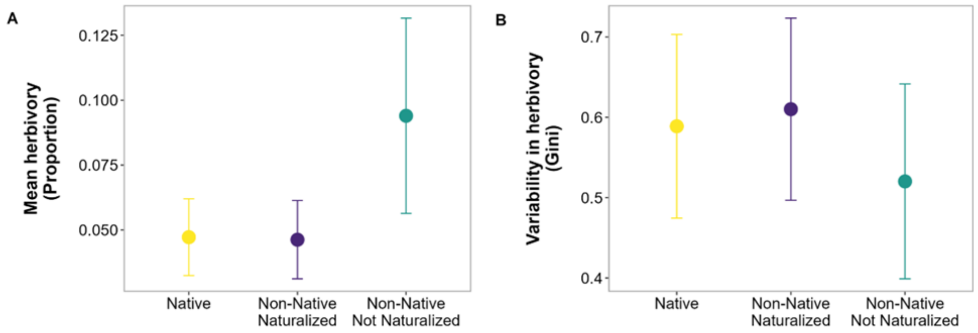

Figure S1: Results from the global analysis of enemy release showing no differences in (a) mean herbivory and (b) variability in herbivory (Gini coefficient) between native (yellow), naturalized (purple) and not naturalized (green) non-native species. Dots show predicted means and lines 95% credible intervals from Bayesian phylogenetic beta regressions.

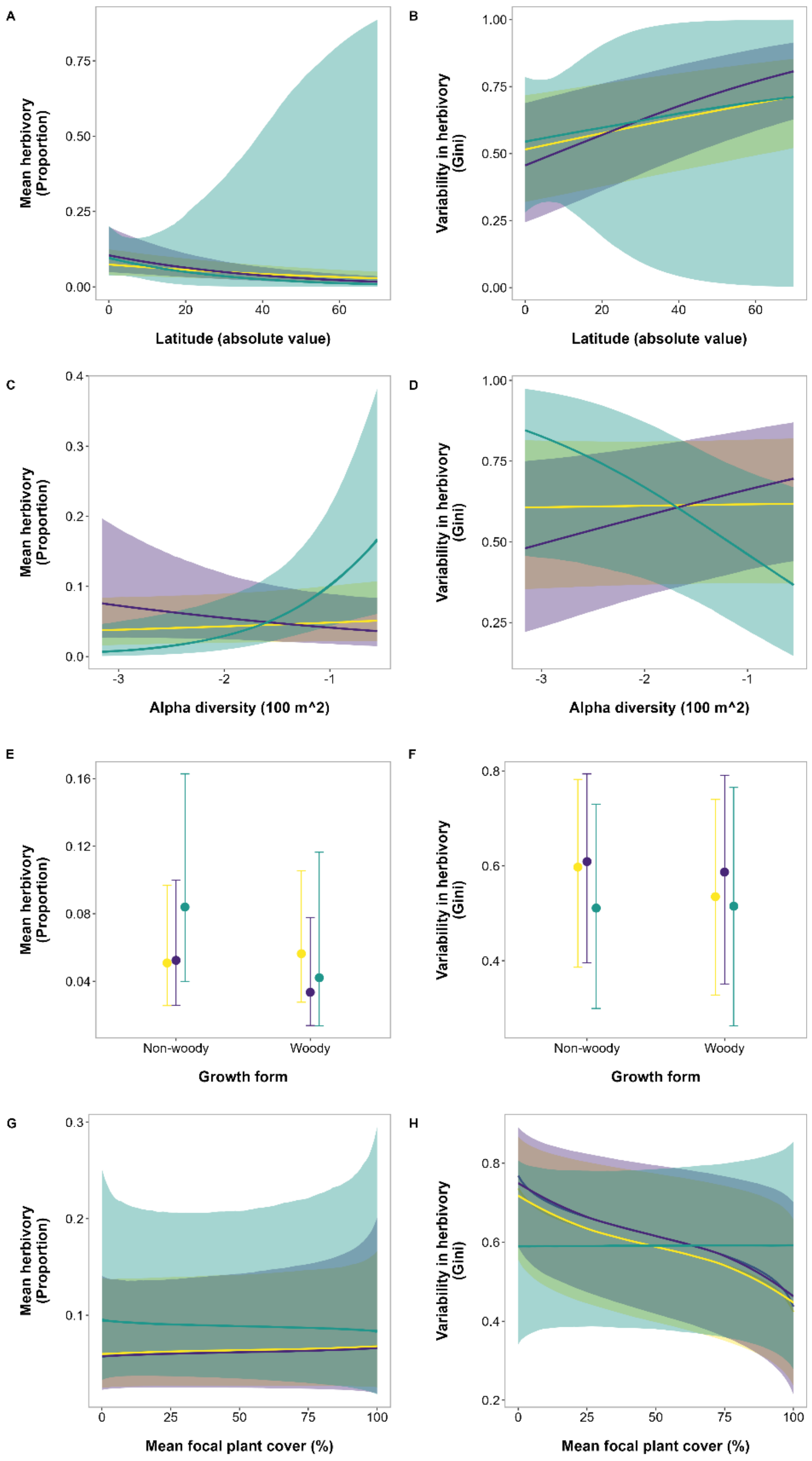

Figure S2: Results from the analyses of ecological factors potentially driving differences in herbivory for native (yellow), naturalized (purple) and not naturalized (green) non-native species. There were no differences in mean herbivory or herbivory variability between native and naturalized or not naturalized non-native populations at different latitudes (a, b), different levels of plant alpha diversity (c, d), nor considering different growth forms (e, f) or with different focal plant cover (g, h). Graphs show predicted means and 95% credible intervals from Bayesian phylogenetic beta regressions.

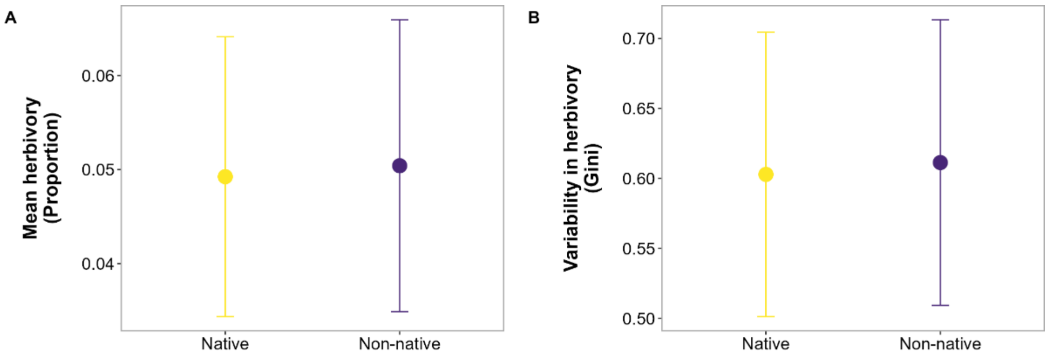

Figure S3: Results from the global analysis of enemy release showing no differences in (a) mean herbivory and (b) variability in herbivory (Gini coefficient) between native (yellow) and non-native (purple) species. Dots show predicted means and lines 95% credible intervals from Bayesian phylogenetic beta regressions.

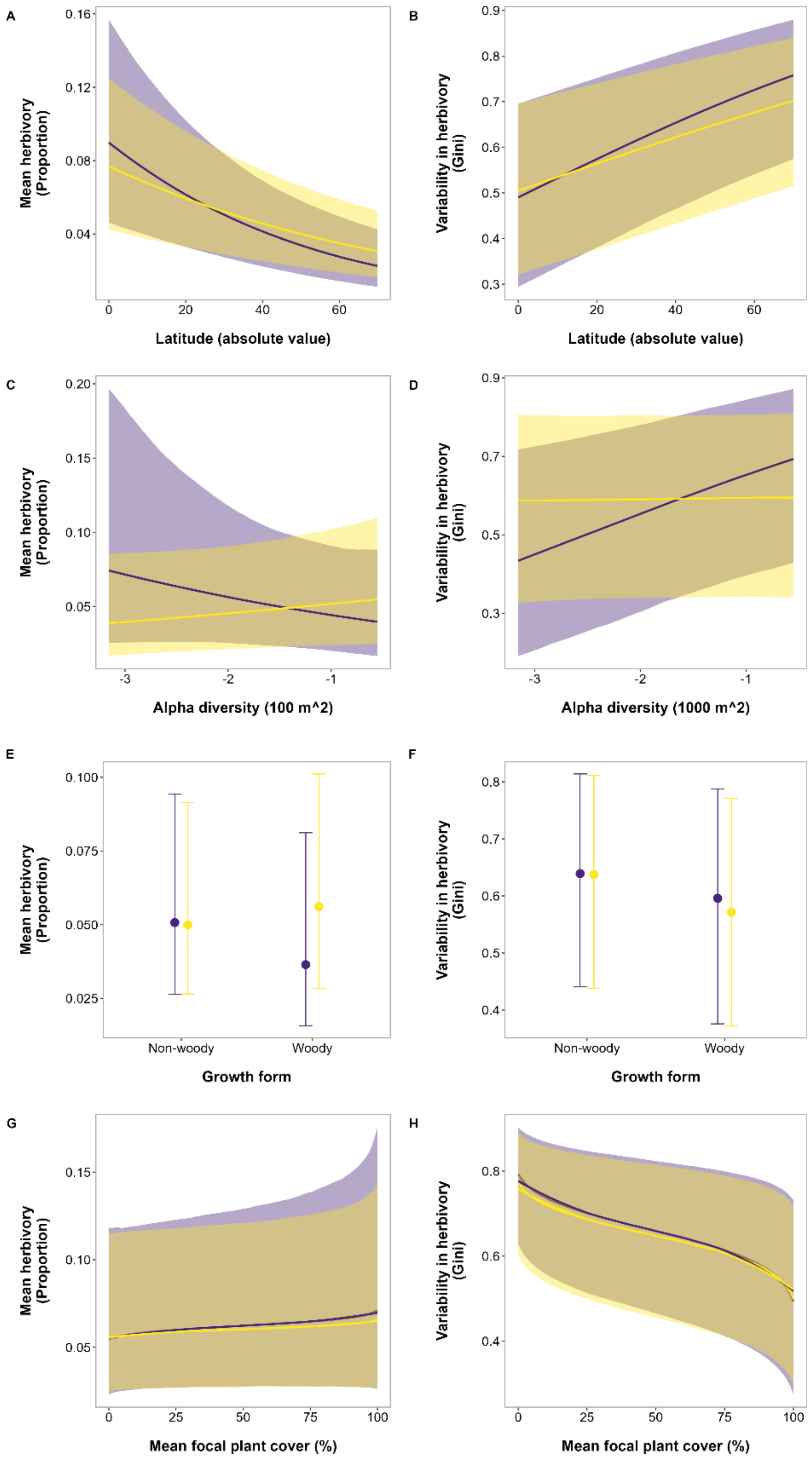

Figure S4: Results from the analyses of ecological factors potentially driving differences in herbivory for native (yellow) and non-native (purple) species. There were no differences in mean herbivory or herbivory variability between native and naturalized or not naturalized non-native populations at different latitudes (a, b), different levels of plant alpha diversity (c, d), nor considering different growth forms (e, f) or with different focal plant cover (g, h). Graphs show predicted means and 95% credible intervals from Bayesian phylogenetic beta regressions.

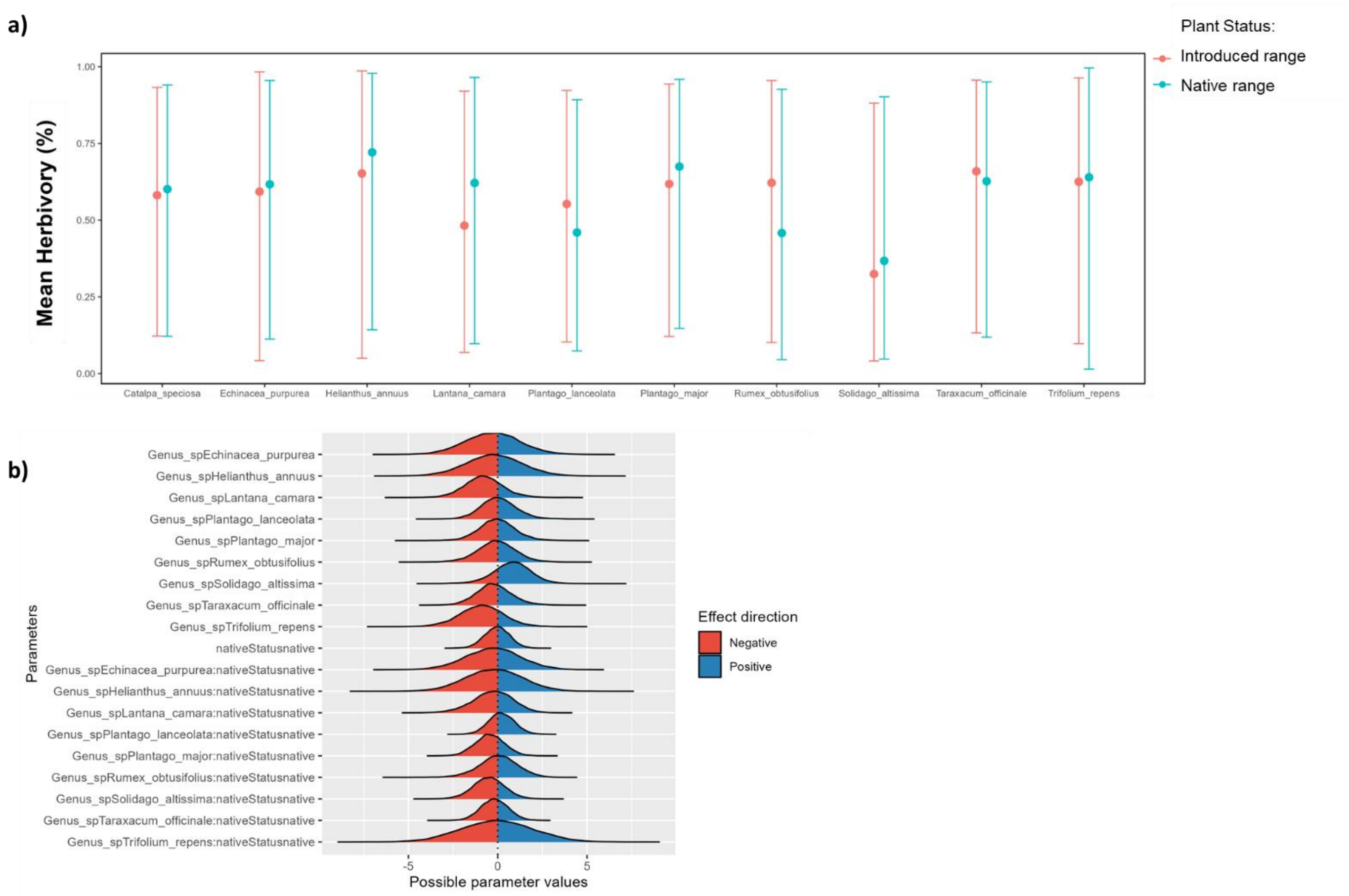

Figure S5: Results from models for the biogeographical subsets of ten species for mean herbivory. a) Comparison of mean intrapopulation variability in herbivory for populations in their introduced (in red) and native (in blue) range for the subset of 10 species included in the biogeographic analysis and b) evaluation plot with the proportion of the posterior distribution of the factors in the model.

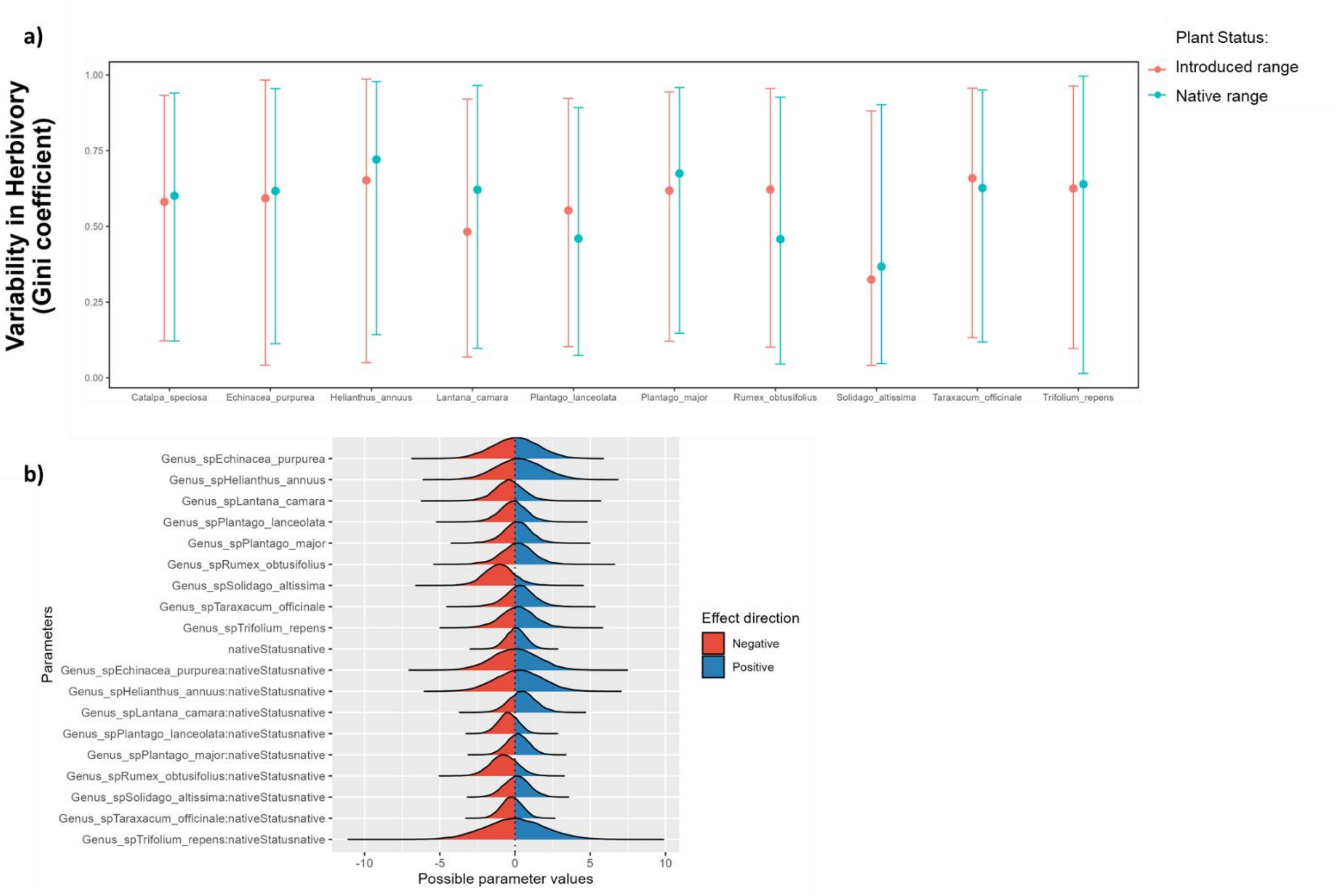

Figure S6: Results from models for the biogeographical subsets of ten species for variability in herbivory (Gini coefficient). a) Comparison of mean intrapopulation variability in herbivory for populations in their introduced (in red) and native (in blue) range for the subset of 10 species included in the biogeographic analysis and b) evaluation plot with the proportion of the posterior distribution of the factors in the model.

a)

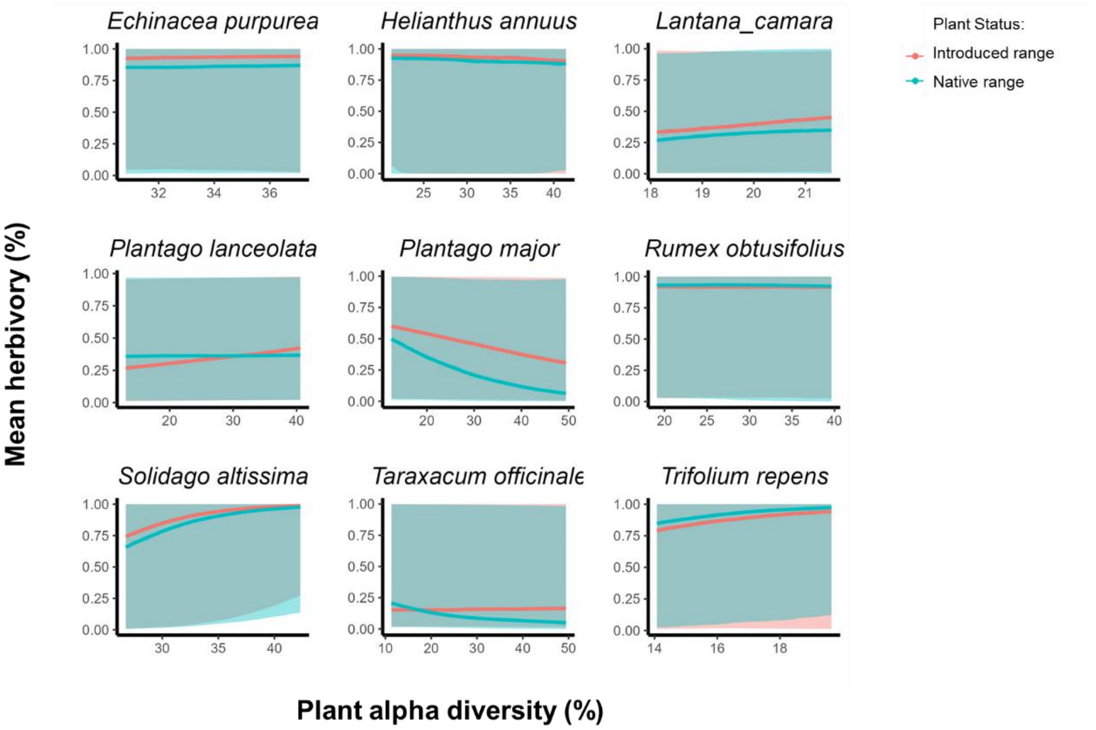

b)

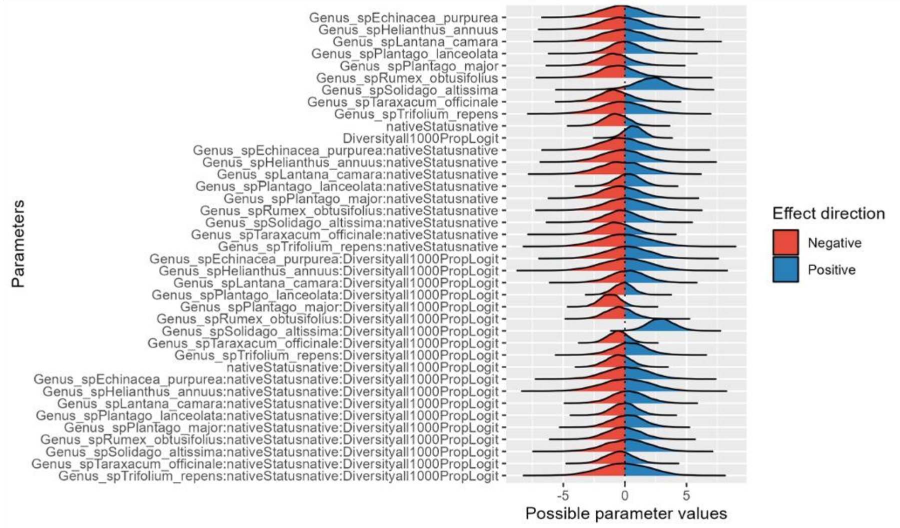

Figure S7: Results from models for the biogeographical subsets of nine species for the interaction between mean herbivory and plant diversity. a) Relationship between mean herbivory and surrounding plant alpha diversity for populations in their introduced (in red) and native (in blue) range for the subset of nine species included in the biogeographic analysis and b) evaluation plot with the proportion of the posterior distribution.

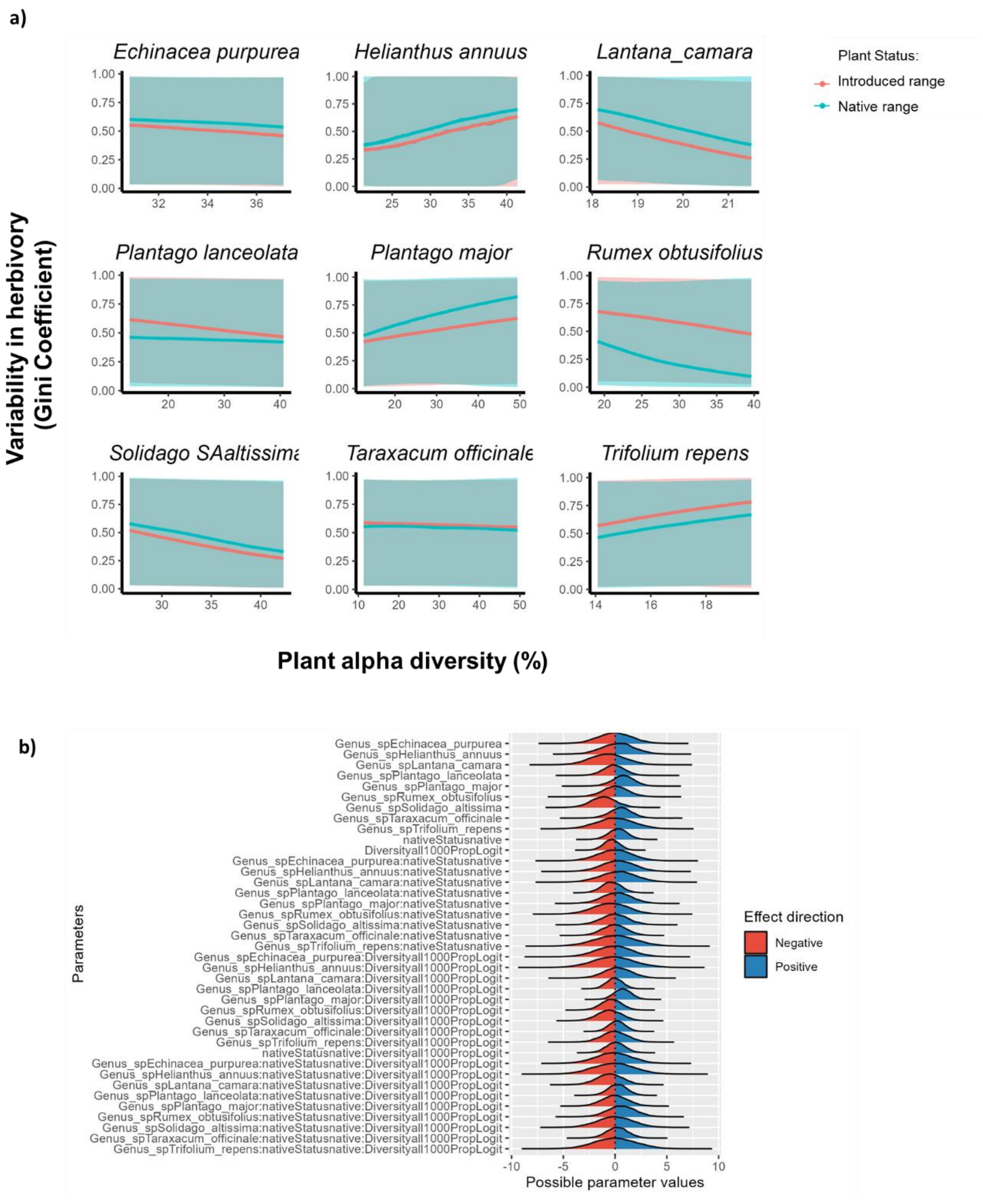

Figure S8: Results from models for the biogeographical subsets of nine species for the interaction between variability in herbivory (Gini coefficient) and plant diversity. a) Relationship between variability in herbivory and surrounding plant alpha diversity for populations in their introduced (in red) and native (in blue) range for the subset of nine species included in the biogeographic analysis and b) evaluation plot with the proportion of the posterior distribution.

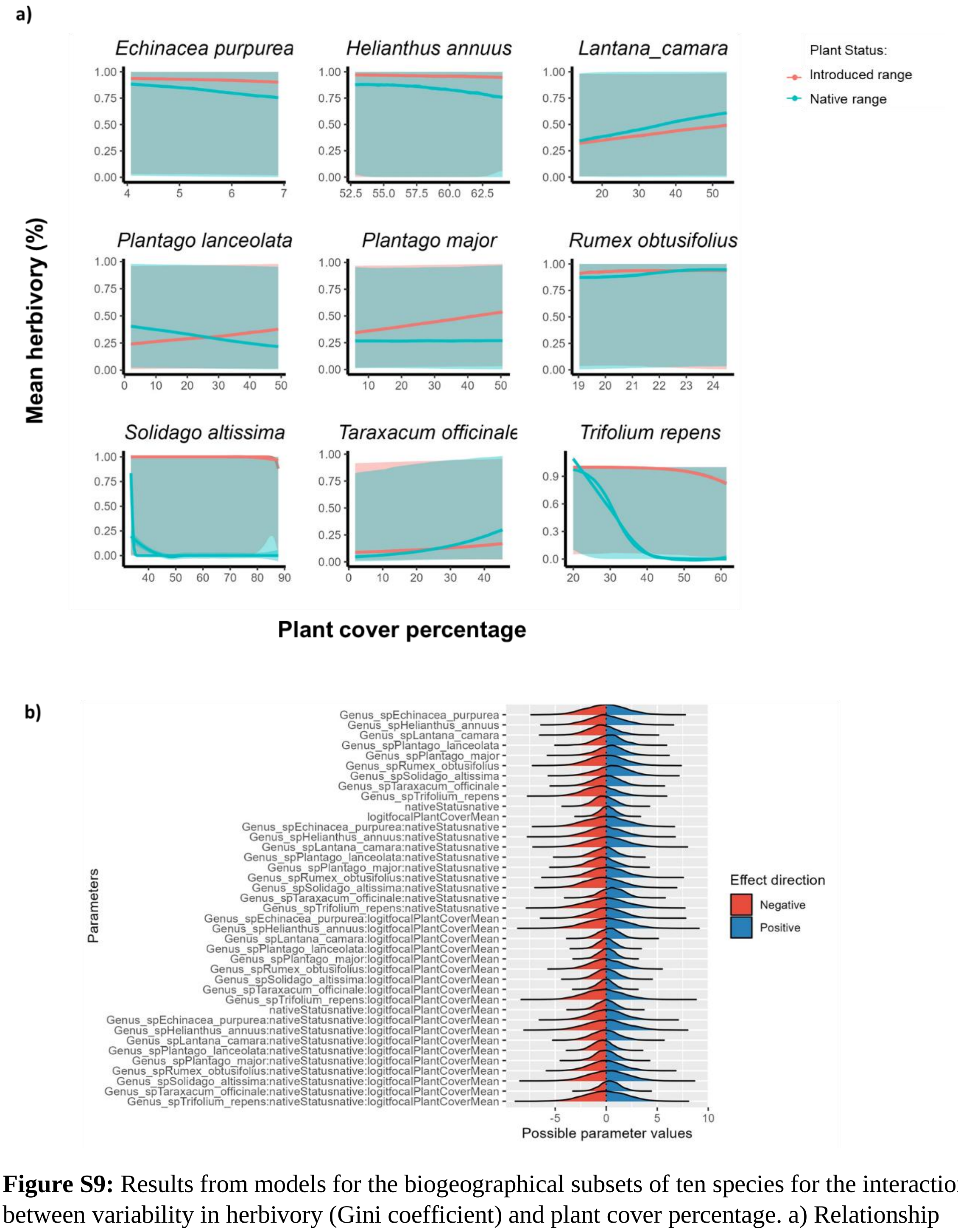

Figure S9: Results from models for the biogeographical subsets of ten species for the interaction between variability in herbivory (Gini coefficient) and plant cover percentage. a) Relationship between variability in herbivory and mean plant cover percentage for populations in their introduced (in red) and native (in blue) range for the subset of nine species included in the biogeographic analysis and b) evaluation plot with the proportion of the posterior distribution.

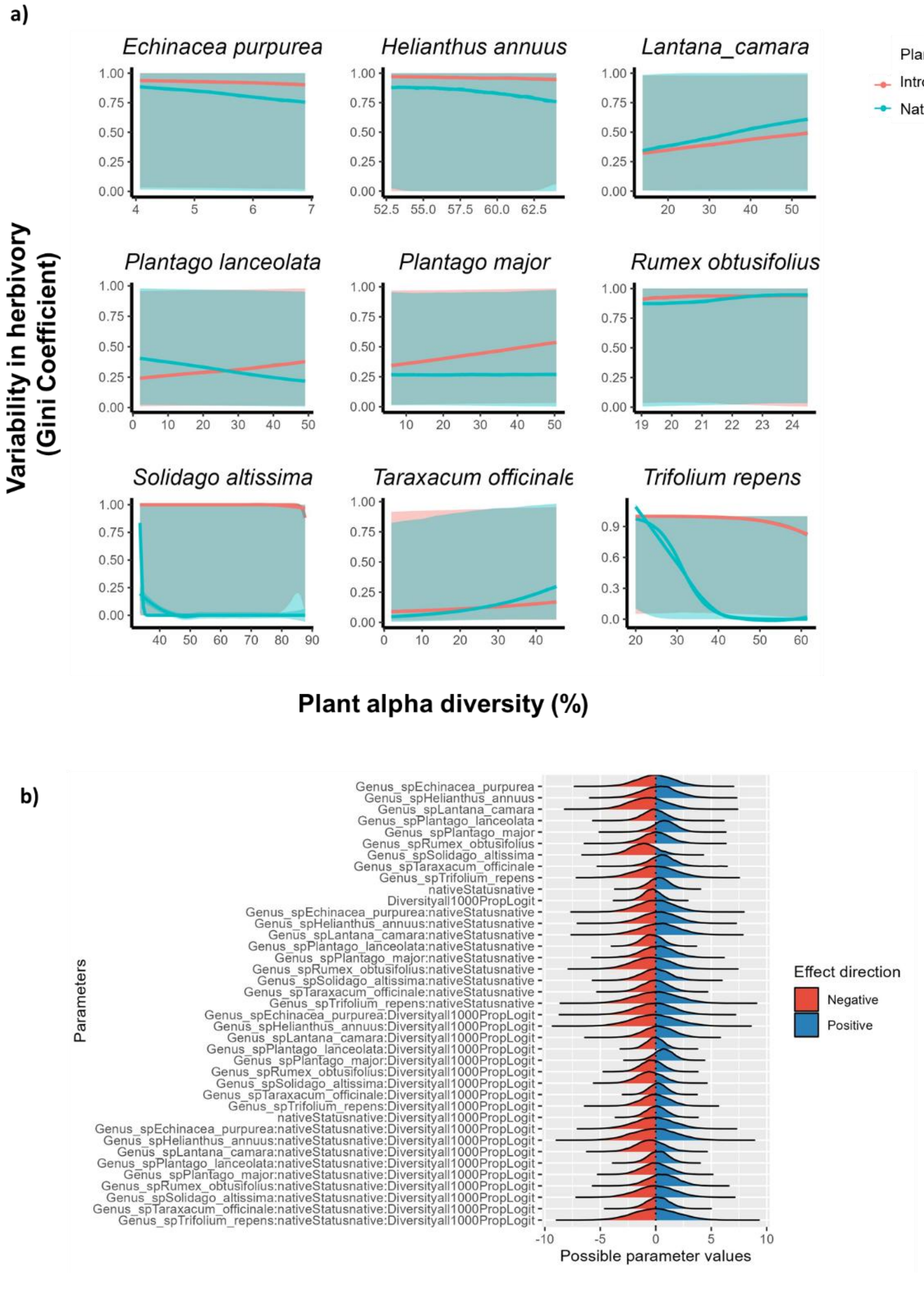

Figure S10: Results from models for the biogeographical subsets of ten species for the interaction between variability in herbivory (Gini coefficient) and plant cover percentage. a) Relationship between variability in herbivory and mean plant cover percentage for populations in their introduced (in red) and native (in blue) range for the subset of nine species included in the biogeographic analysis and b) evaluation plot with the proportion of the posterior distribution.

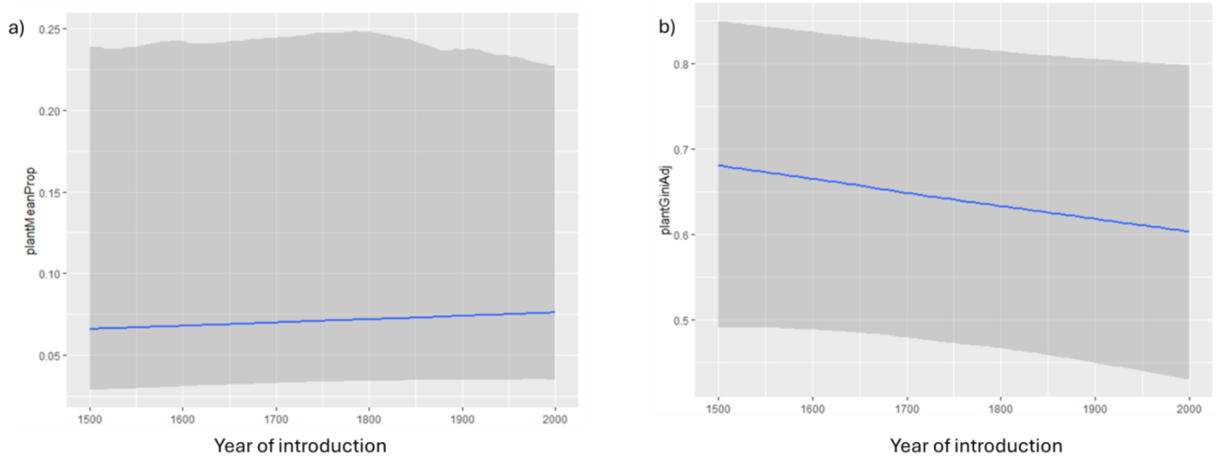

Figure S11: Results from models testing the effect of time since introduction on a) mean herbivory and b) herbivory variability for non native species populations.

Table S1: Non-native species included in the study, indicating their naturalized status (naturalized or not naturalized), their invasive status (invasive or not), and the first year the species was observed in their introduced range country. We search for information about the naturalized status in the Global Naturalized Alien Flora (GloNAF; van Kleunen et al., 2019), for the invasive status we searched in the CABI Digital Library (https://www.cabidigitallibrary.org/) or in regional online repositories. For information on the introduction time, we checked the year of the first observation in the region in the Global Biodiversity Information Facility ("GBIF year"; GBIF; https://www.gbif.org/); second, we performed a literature search for the year of introduction for all different species in the regions where they were sampled ("Year introduction").

**Genus_sp**	**Country**	**Naturalized Status**	**Invasive_Status**	**GBIF year**	**Year introduction**
Abutilon_theophrasti	United States	naturalized	invasive	1824	1750
Acer_campestre	United States	naturalized	invasive	1880	NA
Ageratum_conyzoides	India	naturalized	invasive	1930	1832
Ageratum_fastigiatum	Brazil	not naturalized	NA	NA	NA
Ailanthus_altissima	United States	naturalized	invasive	1942	1784
Alcea_rosea	United States	naturalized	invasive	1828	1800
Alliaria_petiolata	United States	naturalized	invasive	1871	1868
Alternanthera_brasiliana	Nigeria	naturalized	invasive	1996	1890
Alternanthera_ficoidea	India	naturalized	invasive	2000	NA
Alternanthera_sessilis	Nigeria	not naturalized	not invasive	1891	1963
Ammophila_breviligulata	United States	not naturalized	invasive	1836	1890
Andropogon_virginicus	Japan	naturalized	invasive	1940	1940
Arctium_minus	United States	naturalized	invasive	1822	1600
Argemone_mexicana	India	naturalized	invasive	1890	1800
Asclepias_curassavica	United States	naturalized	invasive	1788	NA
Barbarea_vulgaris	United States	naturalized	invasive	1824	1600
Brassica_oleracea	United States	naturalized	not invasive	1824	NA
Calotropis_procera	India	naturalized	invasive	1955	1864
Calotropis_procera	South Africa	naturalized	invasive	2001	1890

Campanula_persicifolia	United States	naturalized	not invasive	1907	NA
Canna_indica	South Africa	naturalized	invasive	1931	1929
Catalpa_speciosa	United States	naturalized	NA	1879	NA
Centaurea_stoebe	United States	naturalized	invasive	1903	1890
Centaurea_stoebe	Germany	naturalized	invasive	1700	NA
Chromolaena_odorata	Nigeria	not naturalized	invasive	1962	1937
Coffea_arabica	Panama	not naturalized	not invasive	1960	1780
Corylus_avellana	New Zealand	not naturalized	not invasive	1949	1800
Crotalaria_juncea	United States	naturalized	not invasive	1923	1930
Crotalaria_pallida	Brazil	naturalized	invasive	1826	NA
Desmodium_incanum	South Africa	not naturalized	invasive	1884	1775
Desmodium_podocarpum	Japan	not naturalized	NA	1910	NA
Echinacea_purpurea	United States	naturalized	not invasive	1815	NA
Elymus_hystrix	United States	naturalized	not invasive	1828	NA
Erigeron_canadensis	Finland	not naturalized	invasive	1878	1878
Euphorbia_esula	United States	not naturalized	invasive	1866	1827
Galium_circaezans	United States	naturalized	NA	1802	NA
Ginkgo_biloba	United States	naturalized	not invasive	1885	1784
Gleditsia_triacanthos	Argentina	naturalized	invasive	1900	1899
Glycine_max	United States	naturalized	not invasive	1896	1765
Helianthus_annuus	United States	naturalized	not invasive	1830	NA
Heliotropium_indicum	Mozambique	not naturalized	NA	NA	NA
Heracleum_sphondylium	United Kingdom	naturalized	NA	NA	NA
Heterotheca_subaxillaris	Israel	naturalized	invasive	1980	1970
Hypochaeris_radicata	Australia	naturalized	invasive	1771	1771
Hypochaeris_radicata	Japan	naturalized	invasive	1943	1933
Hyptis_suaveolens	India	naturalized	invasive	1948	1980
Impatiens_parviflora	United Kingdom	naturalized	NA	NA	NA
Impatiens_parviflora	Canada	naturalized	not invasive	1887	NA
Imperata_cylindrica	Japan	not naturalized	NA	1855	NA
Kuhnia_eupatorioides	United States	not naturalized	not invasive	1815	NA
Lantana_camara	India	naturalized	invasive	1906	1809
Lantana_camara	Portugal	naturalized	invasive	1987	1934
Lepidium_draba	United States	naturalized	invasive	1878	1900
Lespedeza_hirta	United States	not naturalized	not invasive	1801	NA
Lupinus_polyphyllus	Finland	naturalized	invasive	1917	1800
Lythrum_salicaria	United States	naturalized	invasive	1822	1800
Melanthera_scandens	Nigeria	not naturalized	NA	NA	NA
Melilotus_albus	United States	naturalized	invasive	1839	1664
Morus_alba	United States	naturalized	invasive	1807	1600
Nandina_domestica	United States	naturalized	invasive	1879	1800
Oenothera_biennis	Japan	naturalized	not invasive	1919	1920
Olea_europaea	New Zealand	naturalized	NA	NA	NA
Pastinaca_sativa	United States	naturalized	invasive	1823	1600
Paulownia_tomentosa	India	not naturalized	not invasive	2020	1800
Pinus_contorta	Chile	not naturalized	invasive	2013	1970
Plantago_lanceolata	Australia	naturalized	invasive	1772	1801
Plantago_lanceolata	Canada	naturalized	invasive	1828	1829
Plantago_lanceolata	Japan	naturalized	invasive	1919	1850
Plantago_lanceolata	United States	naturalized	invasive	1822	1922
Plantago_lanceolata	Portugal	naturalized	invasive	1955	1968

Plantago_lanceolata	New Zealand	naturalized	invasive	1880	NA
Plantago_lanceolata	Ecuador	not naturalized	not invasive	1963	NA
Plantago_major	United States	naturalized	invasive	1823	1600
Plantago_major	New Zealand	naturalized	invasive	1979	1888
Plantago_major	Argentina	naturalized	invasive	1900	NA
Plantago_major	India	not naturalized	invasive	1924	NA
Plantago_major	Ecuador	not naturalized	invasive	1947	NA
Plantago_major	Panama	not naturalized	not invasive	1893	NA
Potentilla_recta	United States	naturalized	invasive	1808	1900
Ricinus_communis	Panama	not naturalized	NA	NA	NA
Roystonea_regia	India	not naturalized	not invasive	1965	NA
Ruellia_nudiflora	Mexico	not naturalized	NA	NA	NA
Ruellia_tuberosa	India	naturalized	invasive	1956	NA
Rumex_crispus	United States	naturalized	invasive	1823	1700
Rumex_obtusifolius	Canada	naturalized	invasive	1821	1821
Rumex_obtusifolius	Panama	not naturalized	NA	NA	NA
Salpichroa_origanifolia	Portugal	naturalized	invasive	1988	1937
Senecio_elegans	New Zealand	naturalized	NA	NA	NA
Senecio_madagascariensis	Colombia	naturalized	invasive	1986	1980
Senna_obtusifolia	India	naturalized	not invasive	1975	1800
Senna_occidentalis	India	naturalized	invasive	1696	NA
Solanum_dulcamara	United States	naturalized	invasive	1824	NA
Solanum_ptychanthum	United States	not naturalized	not invasive	1864	NA
Solanum_sp	Peru	not naturalized	NA	NA	NA
Solidago_altissima	Japan	naturalized	invasive	1937	1900
Symphyotrichum_novi-belgii	Japan	naturalized	invasive	1947	NA
Taraxacum_officinale	Colombia	naturalized	invasive	1937	1500
Taraxacum_officinale	United States	naturalized	invasive	1830	1672
Taraxacum_officinale	New Zealand	naturalized	invasive	1872	1852
Taraxacum_officinale	Chile	naturalized	invasive	1959	1882
Taraxacum_officinale	Japan	naturalized	invasive	1932	1904
Taraxacum_officinale	Argentina	naturalized	invasive	1885	NA
Taraxacum_officinale	Mexico	naturalized	not invasive	1894	1500
Taraxacum_officinale	Ecuador	naturalized	not invasive	1968	1500
Taraxacum_officinale	Australia	naturalized	not invasive	1973	1769
Tradescantia_fluminensis	Portugal	naturalized	invasive	1987	1894
Triclisia_gilletii	Nigeria	not naturalized	NA	NA	NA
Trifolium_repens	Portugal	not naturalized	NA	NA	NA
Urena_lobata	India	not naturalized	invasive	1835	1827
Verbascum_thapsus	United States	naturalized	invasive	1819	1750
Verbesina_encelioides	Israel	naturalized	not invasive	1980	NA
Vicia_sativa	United States	naturalized	invasive	NA	NA
Vicia_villosa	United States	naturalized	invasive	1802	NA
Zea_mays	United States	naturalized	not invasive	1838	1500
Zea_mays	Belgium	not naturalized	not invasive	1856	1950

Table S2: subset of ten species in their native and introduced ranges indicating the occurrence country and the number of populations on each range and country.

Species name	Country	Plant status	n Populations
Catalpa_speciosa	United States	introduced	3
Catalpa_speciosa	United States	native	1
Echinacea_purpurea	United States	introduced	4
Echinacea_purpurea	United States	native	2
Helianthus_annuus	United States	introduced	1
Helianthus_annuus	United States	native	1
Lantana_camara	Colombia	native	1
Lantana_camara	India	introduced	2
Lantana_camara	Portugal	introduced	1
Plantago_lanceolata	Australia	introduced	1
Plantago_lanceolata	Canada	introduced	1
Plantago_lanceolata	China	native	1
Plantago_lanceolata	Ecuador	introduced	1
Plantago_lanceolata	Finland	native	1
Plantago_lanceolata	Germany	native	4
Plantago_lanceolata	Ireland	native	3
Plantago_lanceolata	Japan	introduced	2
Plantago_lanceolata	New Zealand	introduced	4
Plantago_lanceolata	Portugal	introduced	1
Plantago_lanceolata	Spain	native	8
Plantago_lanceolata	Sweden	native	4
Plantago_lanceolata	Switzerland	native	12
Plantago_lanceolata	United Kingdom	native	1
Plantago_lanceolata	United States	introduced	15
Plantago_lanceolata	United States	native	1
Plantago_major	Argentina	introduced	1
Plantago_major	China	native	1
Plantago_major	Ecuador	introduced	1
Plantago_major	Estonia	native	1
Plantago_major	Finland	native	2
Plantago_major	India	introduced	3
Plantago_major	New Zealand	introduced	1
Plantago_major	Panama	introduced	1
Plantago_major	United States	introduced	8
Rumex_obtusifolius	Canada	introduced	1
Rumex_obtusifolius	Panama	introduced	1
Rumex_obtusifolius	Spain	native	2
Solidago_altissima	Japan	introduced	3
Solidago_altissima	United States	native	2
Taraxacum_officinale	Argentina	introduced	4
Taraxacum_officinale	Australia	introduced	3
Taraxacum_officinale	Chile	introduced	2

Taraxacum_officinale	Colombia	introduced	1
Taraxacum_officinale	Ecuador	introduced	1
Taraxacum_officinale	Finland	native	2
Taraxacum_officinale	Germany	native	7
Taraxacum_officinale	Japan	introduced	1
Taraxacum_officinale	Mexico	introduced	1
Taraxacum_officinale	New Zealand	introduced	2
Taraxacum_officinale	Norway	native	1
Taraxacum_officinale	Spain	native	1
Taraxacum_officinale	Switzerland	native	2
Taraxacum_officinale	United Kingdom	native	1
Taraxacum_officinale	United States	introduced	8
Trifolium_repens	Germany	native	1
Trifolium_repens	Portugal	introduced	1
Trifolium_repens	United Kingdom	native	1

SAMPLING PROTOCOL ACCESSIBILITY:

The primary and alternative protocols are available on the HerbVar website: https://herbvar.org/protocols.html
